# Effect of Oral Ingestion of Low-Molecular Collagen Peptides Derived from Skate (*Raja Kenojei*) Skin on Body Fat in Overweight Adults: A Randomized, Double-Blind, Placebo-Controlled Trial

**DOI:** 10.3390/md17030157

**Published:** 2019-03-07

**Authors:** Young Jin Tak, Yun Jin Kim, Jeong Gyu Lee, Yu-Hyun Yi, Young Hye Cho, Geun Hee Kang, Sang Yeoup Lee

**Affiliations:** 1 Department of Family Medicine, Pusan National University School of Medicine, Busandaehak-ro, Mulgeum-eup, Yangsan-si 50612, Korea; 03141998@hanmail.net (Y.J.T.); yujkim@pusan.ac.kr (Y.J.K.); jeklee@pnu.edu (J.G.L.); eeugus@gmail.com (Y.-H.Y.); 2 Biomedical Research Institute, Pusan National University Hospital, Busan 602-739, Korea; 3 Family Medicine Clinic and Research Institute of Convergence of Biomedical Science and Technology, Pusan National University Yangsan Hospital, Yangsan 50612, Korea; younghye82@naver.com; 4 Yeong San Skate Co., Ltd., Naju 58266, Korea; skate1438@naver.com; 5 Medical Education Unit, Pusan National University School of Medicine, Yangsan 50612, Korea

**Keywords:** Skate skin, *Raja kenojei*, collagen, body fat, obesity

## Abstract

Recent animal studies found the potential of a collagen peptide derived from skate skin to have anti-obesity effects through the suppression of fat accumulation and regulation of lipid metabolism. However, no studies have yet been performed in humans. Here, this very first human randomized, placebo-controlled, and double-blinded study was designed to investigate the efficacy and tolerability of skate skin collagen peptides (SCP) for the reduction of body fat in overweight adults. Ninety healthy volunteers (17 men) aged 41.2 ± 10.4 years with a mean body mass index of 25.6 ± 1.9 kg/m^2^ were assigned to the intervention group (IG), which received 2000 mg of SCP per day or to the control group (CG) given the placebo for 12 weeks and 81 (90%) participants completed the study. Changes in body fat were evaluated using dual energy X-ray absorptiometry as a primary efficacy endpoint. After 12 weeks of the trial, the percentage of body fat and body fat mass (kg) in IG were found to be significantly better than those of subjects in CG (−1.2% vs. 2.7%, *p* = 0.024 and −1.2 kg vs. 0.3 kg, *p* = 0.025). Application of SCP was well tolerated and no notable adverse effect was reported from both groups. These results suggest the beneficial potential of SCP in the reduction of body fat in overweight adults.

## 1. Introduction

The global burden of cardiovascular diseases (CVD), which is the most common cause of death is increasing rapidly despite tremendous treatment options for diabetes, hypertension and dyslipidemia [1]. It is believed to be mainly attributed to failing to control the growing number of populations who are obese as a sedentary lifestyle and westernized eating habits have become prevalent worldwide [2]. Obesity is the root cause of chronic diseases that have been afflicting about one-third of the total population globally and the crucial factor to solve for prevention of fatal health issues [3]. Although medical experts have been struggling to find out effective treatment options for obese people, there have been only a few medications used to suppress appetite by working on the central neurologic system, which often brings about unexpected side effects such as the urge of suicide, aggravation of depression or other psychological issues [4]. Therefore, it is necessary to discover a material with an anti-adipogenic effect to help lose weight that comes from naturally available sources without any concerns of side effects. 

In an attempt to make these possible, numerous products are now being developed and commercialized. Among them is collagen that has been widely used as a material in food, cosmetic, and pharmaceutical industries due to its biological and functional properties [5]. In a recent randomized controlled trial, post-exercise supplementation of a collagen peptide in combination with resistance training was seen to improve body composition and increase muscle strength in 148 elderly sarcopenic men [6]. Especially, collagen peptides derived from sea fish have been recognized as a dietary supplement which is beneficial to blood pressure [7] and glucose control [8], skin moisturizing [9] and improved lipids [10]. 

Skate is quite a popular food consumed widely in Korea in both a raw and fermented form. From a nutritional perspective, skate is known to contain taurine, which plays a role in the growth and development of cells and is an energy booster, anserine that acts as a buffer for muscles [11]. In addition, it is high in essential fatty acids such as linoleic and linolenic acid, which are beneficial to enhance cognitive functions by reducing a high lipid level [12]. As many healthy nutrients that skate contains are discovered [13,14], its demand has been on the rise in recent years. Accordingly, a great number of by-products such as skin and cartilage remain and go waste in the process of the meat part of the skate. However, the nutritional values of by-products from skate are also reported to be fairly high [15], so these can be a useful resource in the fish-supplements industry [16,17]. Previous information suggests that skate can serve as a good nutrition source for the development of highly functional collagen peptide materials [11].

In very recent study, Woo et al. found the anti-obesity effects of collage peptide derived from skate skin by suppressing fat accumulation and regulation of lipid metabolism in genetic and high-fat diet (HFD)-induced obese animal model [18], presenting that using skate skin might be an effective and safe approach for humans to resolve obesity-related health problems. Although an HFD-induced obese animal is considered to have similar pathophysiology to that of an obese person [19], a well-designed clinical trial is necessary to confirm the effect and safety of skate skin collagen peptide (SCP) in humans. However, the effects of collagen from skate skin on changes in body weight have never been explored in humans. Thus, our study aimed to assess the efficacy and tolerability of SCP for the reduction of excess body fat in overweight adults. We hypothesized that SCP is an effective, safe agent for the treatment of obese people. 

## 2. Results

### 2.1. General Characteristics of the Study Subjects

Five participants in IG and four in CG withdrew consent for personal reasons that were not considered associated with the trial. The characteristics of these nine participants were similar to ones of the others who completed the study. Compliance was satisfactory with participants taking more than 90% of the supplements given in both IG and CG. Randomization was successful, as the two groups generated were comparable for most variables and no significant differences were observed in the baseline demographic and anthropometric characteristics between the two groups (Table 1). The majority of the participants were women (81.1%) and the mean age of total participants was 41.2 ± 10.4 years. The average BMI of both IG and CG was over 25.6 ± 1.9 kg/m^2^, which is more than the cutoff for defining obesity in Korea [20]. Furthermore, no statistically significant intergroup differences were observed for alcohol drinking, smoking, calorie intake and physical activities at baseline. During the whole study period, the double-blind requirement was well maintained.

### 2.2. Changes inBody Composition

**Figure 1:**
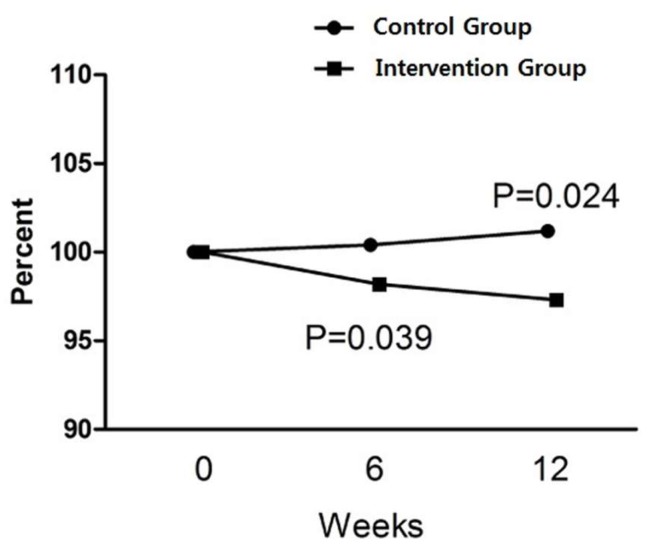
The percentage of changes in body fat during the 12 weeks of the study. *P*-values were derived from Mann-Whitney’s U test with the intention to treat analysis (*n* = 90).

There were no significant changes in calorie intake (Δ IG: 133.1 kcal/day, *p* = 0.715 vs. Δ CG: −51.7 kcal/day, *p* = 0.436) and physical activities (Δ IG: −205.6 METs, *p* = 0.166 vs. Δ CG: 129.4 METs, *p* = 0.551) checked at baseline and the 12th week of the trial among the participants, reflecting no additional effect that might have influenced body composition aside from the intervention. After 12 weeks of the trial, CG subjects showed a slight rise in BW by 0.7 kg (*p* = 0.018), and accordingly BMI by 0.3 kg/m^2^ (*p* = 0.015), while IG participants had no significant increase in BW and BMI (Table 2). However, BF in IG after 12 weeks was found to decline (−0.6%, *p* = 0.017) from the baseline with a little decrease of lean body mass. In contrast, there was no change in body composition among CG subjects. In terms of comparison between the groups after 12 weeks, IG subjects turned out to have more body fat loss than the CG ones (−1.2 kg vs. 0.3 kg, *p* = 0.025). This intergroup difference in the percentage of BW change was observed from the sixth week of the trial and lasted until the 12th week (Figure 1).

### 2.3. Changes in Laboratory Measurements

In both groups, adiponectin levels rose a bit with a larger increase found in IG although it was not a significant difference between the groups after 12 weeks. When it comes to the lipid profile, no changes were observed except for n aHDL-C decrease in IG.

### 2.4. Safety

Most of the subjects completed the protocol without adverse symptoms. One subject in IG complained of dyspepsia and decided to withdraw consent for that reason. However, this symptom was not determined to have anything to do with taking SCP. No clinical changes in the levels of liver enzyme, creatinine and glucose were observed in each group. No intergroup differences in these figures were found during the study period (Table 3). 

## 3. Discussion

To our best knowledge, this study is the first randomized, double-blind, placebo-controlled trial to identify the efficacy, safety, and tolerability of SCP in overweight people. Although the molecular mechanism underlying the anti-obesity effect of fish collagen peptides has now been discovered, the influence of them on body fat in humans had yet to been addressed before our study. Thus, we aimed to see whether SCP could reduce human body fat as well. Our results showed that oral SCP of 2000 mg daily for 12 weeks decreased a small amount of body fat and was tolerated without reducing calorie intake and increasing physical activities. 

The biological underlying mechanism of this current outcome can be found in previous animal studies conducted recently [21,22]. Lee et al. observed in obese mice that the oral administration of fish collagen peptide (FCP) significantly reduced body weight gain induced by HFD without a significant difference in food intake, confirming that FCP has an anti-adipogenic effect in in vitro and in vivo models [21]. Their findings demonstrate that subcritical water-hydrolyzed FCP inhibits lipid accumulation during the differentiation of 3T3-L1 preadipocytes into adipocytes by suppressing the expression of adipogenic master transcription factors such as peroxisome proliferator-activated receptor-γ (PPAR-γ), CCAAT/enhancer binding protein-alpha (C/EBP-α) and adipocyte protein 2 (aP2) genes, which mainly regulate the differentiation and maintenance of adipocytes, leading to a significant decrease in adipocyte size. Moreover, FCP improved the lipid profile showing reduced serum levels of TC, TG, and LDL-C while increased HDL-C. Another study conducted by Astre G et al. confirmed the previous results showing HFD-fed mice supplemented by FCP exhibited a significantly lower weight gain as soon as the 12th week of treatment, whereas no effect was observed in control group mice [22]. Additionally, lower glucose and a decrease of inflammatory cytokines were seen among mice treated with FCD, presenting a potential effect of FCP on insulin sensitivity.

Although Lee et al. [21] and Astre G et al. [22] did not use skate as a source of collagen peptides in their studies, skate skin is also reported as a good source of collagen, consisting of properties of amino acid-rich collagen. The major amino acids of skate skin are arginine, proline, and glutamic acid. Of the total amino acids, approximately 19.8% of arginine and 12% of proline are presented in the skin part [12]. The stability of collagen is proportional to the total amount of collagen and associated with the pyrolidine (proline + hydroxyproline) content [23], thus it can be enhanced by using skate skin. A large amount of skate skin is being disposed of as skate is becoming popular in both the fresh and fermented form owing to the recognition of its high quantity of nutrients and unique flavor in Korea. From an environmental perspective, therefore, it would be a good solution to prevent by-products from being excessively generated.

More recent findings of Woo et al. [18] specifically using collagen peptide derived from skate skin were consistent with the previous research. In this study, a reduced increase in body weight and visceral adipose tissue was observed in the SCD-fed groups compared to the control group. The anti-obesity effects of SCP were attributed to being mediated by regulating transcription factors and enzymes which regulate hepatic lipid metabolism. Histological analysis of the liver revealed that SCP suppressed hepatic lipid accumulation and reduced the lipid droplet size in the adipose tissue. TG-lowering effect of SCP significantly suppressed adipose tissue differentiation in a dose-dependent manner, which was also demonstrated by the histologic results in adipose tissue. Additionally, the researchers observed that the intake of SCP increased the hepatic protein expression of phosphorylated 5’ adenosine monophosphate-activated protein kinase (p-AMPK) with elevated adiponectin and reduced leptin levels. As a regulator of energy balance by affecting whole body fuel utilization, AMPK induces fatty acid oxidation and inhibits adipocyte differentiation and the synthesis of hepatic fatty acid, cholesterol [24] emerging as a key target for obesity resolution. It is also involved in the regulation of adiponectin, which can activate p-AMPK [25]. In line with this result, our study showed a slightly larger increase in the adiponectin level in IG than that in CG; although this difference between the groups was not statistically significant. For now, we cannot determine whether this increase in adiponectin shown in the human study is mediated by the same way that SCP works on p-AMPK in an animal model. However, our finding can be clinical evidence that p-AMPK may be a good target with respect to SCP related human experimental study in the future.

In our study, IG saw no statistically significant difference in fat mass (kg). However, there was a significant change in the body fat percentage (%). Methodologically, body fat (%) by DXA indicates the body fat (kg) normalized by total body weight (body fat (%) = body fat (kg)/total body weight (kg)) [26]. Thus, an increase in total body weight causes underestimated body fat (%) and a decrease in body weight comes with overestimated body fat (%) even though actual body fat is the same. Our data shows that the total body weight of CG after 12 weeks was slightly higher than that of IG in which the difference was not statistically significant. This point may be attributable to the non-significance in fat mass (kg) change.

In contrast to those animal studies, the present study failed to find improvements in lipid profile with a slight HDL-C decrease found in IG. The authors cannot infer a convincing explanation for that from the current findings. Presumably, it was probably because HDL-C is known to mainly increase by boosting physical activity [27], which was not conducted in our study. From a clinical point of view, although there was a statistically significant HDL-C reduction in IG, the absolute amount seems not clinically important since both levels of HLD–C before and after SCD administration were still at a desirable level from a CVD prevention perspective. Additionally, lipid metabolism is much more complicated in humans than in animal models and many other factors may have been involved in the process that our study missed including covariates. Furthermore, our primary outcome was a change in body fat. Therefore, the size of the study might not be large enough to identify the effect of SCD on lipids with a study period of 12 weeks. Most importantly, previous animal studies induced weight gain by feeding a high-fat diet in order for mice to reach a hyperlipidemic state before administration of SCD while the majority of our study subjects had normal lipid levels at the baseline and this study did not apply the intervention to the subjects for an improvement in lipid levels. 

To fight against obesity and related metabolic disorders, several anti-obesity drugs have been approved by the FDA for the treatment of obesity. However, it is unclear whether these medications actually bring about improved health outcomes including the prevention of CVD and improve the quality of life given that trials of medication-based weight loss interventions showed high study drop-out rates (≥35% in half the included trials) and the differences in these outcomes were small among those on medication compared with placebo [28]. Known adverse effects, financial burden by hefty prices of medications, and failure of the significant outcome of losing weight seem to force obese people to seek alternative treatments with fewer side effects. In this context, many natural products have been tested as potential alternative therapies for obesity and our study was one of them. 

Consequently, although the absolute amount was small, body fat loss found in our IG was quite impressive, when considering the fact that the duration of the intervention was relatively short and that the study did not require the participant to engage in any program in order to increase physical activity and cut down on calorie intake so that they could keep their daily routine as they usually had done. Most of the time, subjects who are involved in studies which are designed to demonstrate the efficacy of anti-obesity drugs are provided with dietary advice to decrease calorie intake and are encouraged to participate in moderate physical activity programs at least three times a week, which can be difficult for the subjects to adhere to the trial to the end showing a high drop-out rate and leads to a lower weight loss outcome than expected [29,30]. Additionally, in our study, the reduction in body fat occurred as soon as 6 weeks after taking SCD, showing a significant difference from CG and this gap was sustained until the study ended. On the other hand, recent research has focused on the roles of gastrointestinal peptides in obesity control [31,32]. They are known to be potential regulators of satiety such as cholecystokinin (CCK) and to influence food intake, which is critical when it comes to losing weight [33]. With regards to this point, further study is needed to see if SCD has an impact on gastrointestinal peptides.

The present study has some limitations. We included woman-dominant participants (81%) who are considered relatively better at complying with a study protocol and more conscious about their weight and appearance than men, which may result in a more favorable outcome in weight control intervention. Moreover, since a 24-h dietary recall is affected by day-to-day variation, the one-day investigation may not represent the usual intake of the subjects [34]. The IPAQ also has a substantial measurement error with the tendency to overestimate, although it consists of 27 questions which reflect on the previous seven days’ physical activities [35]. These weaknesses of self-report surveys led to the result of a negative caloric balance shown in our subjects. However, the researchers asked the participants not to try to change their eating and physical patterns throughout the trial except for taking SCD so that we could assess the effect of SCD possibly only. In this context, the main purpose of the 24-h dietary recall and IPAQ in our study was to see if there were significant differences of before and after SCD administration in the overall calorie intake or expenditure in the daily routine of the subjects. Consequently, our data showed no changes between the two cited time points as planned. If there were any changes in them, we would have analyzed data adjusting for the amount of calories that the participants consumed and spent. Lastly, the current study was conducted by a single center, which can be limited to generalize the results. 

Despite these limitations, our study is considerably valuable owing to several strengths. Firstly, to the best of the authors’ knowledge, it is the first clinical study to examine the efficacy and tolerability of SCP on obesity. Moreover, the measurements of body composition were checked by DXA that is more accurate [36] than bioelectrical impedance analysis, which is most commonly used in the clinical study and private clinics due to its lower cost and simplicity to apply [37]. Furthermore, many trials of weight loss interventions have focused on body weight as their primary efficacy endpoint [28]. However, when it comes to resolution of obesity, what is fundamentally important is whether the intervention can effectively reduce body fat, not just body weight. The current study measured the change in body fat by using a reliable quantitative method through DXA. 

We confirmed that there was no toxicity or severe adverse effect when SCP was applied to humans. More importantly, only SCD supplement for three months without having to change dietary habits and physical performance decreased more body fat than the placebo significantly (−1.2 kg vs. +0.3 kg, *p* = 0.025) and its effect came out as soon as six weeks after it was taken whereas no effect was observed in CG. The study suggests that SCD can help reduce excess body fat and it can be considered a potential alternative treatment for obesity itself and associated disorders. If it is combined with exercise and dietary intervention, the effect of SCD can be greater. However, a replicated study with a larger population is needed so as to reconfirm this favorable effect of SCP on body composition and to elucidate the mechanism responsible for the action of SCP in humans. 

## 4. Materials and Methods

### 4.1. Study Design and Study Subjects

The present study was designed as a randomized, placebo-controlled, double-blind controlled clinical trial and approved by the Institutional Review Board at Pusan National University Yangsan Hospital (IRB No. 02-2017-012). We carried out the study in accordance with the principles of the Declaration of Helsinki from 26 June 2017 through 5 June 2018. Written informed consents following a fully detailed description of the study protocol were obtained from all participants before enrollment. This trial is registered with ClinicalTrials.gov Identifier: NCT03409705.

Eligible subjects were overweight, or obese, defined according to the guidelines of the Korean Society for the Study of Obesity [20]. One hundred adults aged between 20 and 60 years with any value from 23 to 30 kg/m^2^ of body mass index (BMI) were enrolled through recruitment posting at a tertiary hospital in Yangsan. The individuals were excluded if they had any conditions as following; (1) previously taken any medication or supplements that can cause a change in body weight within the past one month including anti-depressants, anti-absorptive agents, appetite suppressors and any other hormonal products, (2) history of engagement in commercial anti-obesity programs within the past three months, (3) being treated for hyperthyroidism or hypothyroidism, (4) alcohol abuser, (5) quit smoking within three months of enrollment, (6) uncontrolled blood pressure, blood glucose, or gastrointestinal symptoms, (7) an aspartate aminotransferase (AST) or alanine aminotransferase (ALT) serum level greater than 80 mg/dL or a creatinine (Cr) level greater than 1.5 mg/dL, (8) pregnant or lactating women or 9) allergic to the ingredient involved. In addition, for safety reasons, candidates diagnosed with cardiovascular diseases or any cancer during the six months prior to study commencement were also excluded. Four participants met the exclusion criteria and ten participants declined to participate. 

### 4.2. Randomization

**Figure 2:**
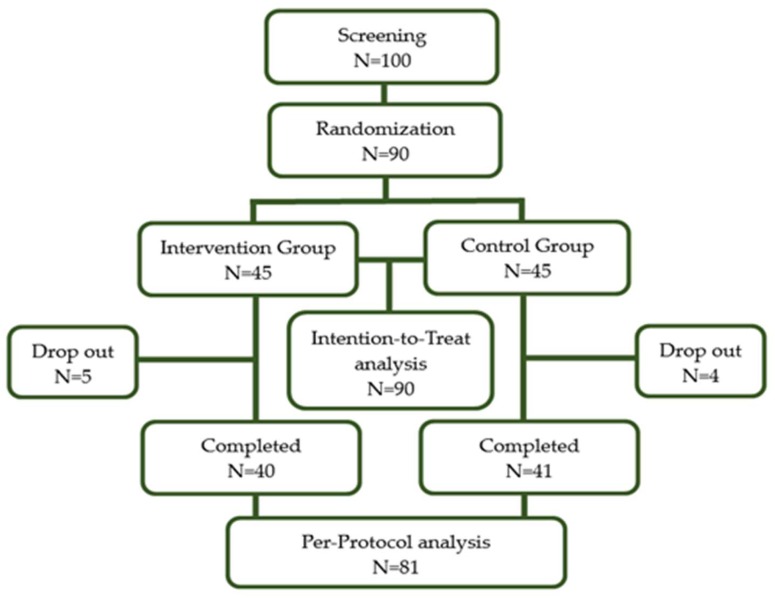
Flow diagram of the study subjects.

One hundred adults were recruited for screening and 90 (90%) participants were finally enrolled. After undergoing baseline measurements, they were randomly assigned to either one of the two groups through block randomization methods using randomized numbers and given identification numbers on recruitment: the intervention group (IG) (*n* = 45), which received 2000 mg of SCP per day in the form of capsules, or the control group (*n* = 45) which was given a placebo (Figure 2). Randomization codes were created by an expert in statistics using nQuery Advisor 7.0. Those who were responsible for deciding on study eligibility and conducting the measurements were kept unaware of the results of the randomization throughout the whole study process. All of the participants were asked to visit the center four times in total (visit 1; for screening, visit 2; randomization and start taking supplements, visit 3; 6 weeks after intervention, visit 4; 12 weeks later).

### 4.3. Intervention

The dosage of SCP applied to the subjects had been determined based on the results from previous animal studies where mice fed 300 mg of SCP daily had shown a significant reduction in body fat without any adverse events. Given the dosage of 300 mg applied to mice of which an average body weight was in these studies [18,19], 2000 mg of SCP was considered appropriate to be given to humans with an average body weight of 60 kg according to the guidance for estimating the maximum safe starting dose in initial clinical trials for therapeutics in adult healthy volunteers [38]. Two capsules (500 mg per capsule) of SCP were taken twice a day in the morning and evening by the subjects in IG (total four capsules each day) for 12 weeks. Subjects in CG were given the placebos with the same protocol and duration. Capsules were visually identical and supplied by Serom Co., Ltd. (Jeonnam, South Korea). Compliance was assessed by counting the remaining capsules at every visit and less than 80% of the taken number of capsules was considered to have dropped out from the study. Reports of any adverse event or unpredicted drug reaction were reported throughout the study. 

### 4.4. Evaluation of Dietary Intake and Physical Activities

At baseline and the 12th week of the trial, the study subjects were asked to answer the questionnaire on dietary intake and physical activities that mainly determine the change in body weight so as to check if there was a significant alteration in their daily routine and to take into account the extra possible effect on their body composition. Dietary intake was investigated by the 24-h recall, which is an open-label nutritional survey method for estimating all food products ingested by the study subjects during the previous 24 h, together with dietary information (time, location, types of food, amount, and cooking method) and hereby reflecting the recent calorie intake of individuals [39,40]. Alcohol drinking was defined as consumption of alcohol with an average of seven cups for men and five or more for women, more than two times a week [41] Frequency, intensity and type of physical activities that the participants had done during the previous weeks were reported using the international physical activity questionnaires (IPAQ) [35] and the number of physical activities were represented as the metabolic equivalent of task (METs). 

### 4.5. Measurements

As the primary outcome was changed into body fat mass (kg) and body fat percent of each subject, dual-energy X-ray absorptiometry (DXA) (Lunar Prodigy 8.50, Lunar Radiation Corp., Madison, WI, USA) was implemented twice at baseline and 12th week of the study. The fat mass percentage was calculated as fat mass/(fat mass + lean mass + bone mineral content). Secondary outcome variables were changes in BMI, sagittal abdomen distance (SAD), fat mass (kg) and lean mass (kg) checked by DXA, lipid profile (triglyceride (TG), total cholesterol (TC), high density lipoprotein cholesterol (HDL-C), low density lipoprotein cholesterol (LDL-C)), free fatty acid (FFA), and adiponectin. The participants were asked to maintain a fasting state for at least 4 h before the test. SAD was measured while the participants were lying supine on their back. A caliper with two sliding arms attached parallel to a vertical scale (Holtain-Kahn Abdominal Caliper 50 cm (98.609XL), U.K.) (Holtain-Kahn Abdominal Caliper Extra Long 50 cm (Holtain Model 609 XL), Seritex Inc, 1 Madison St. East Rutherford, NJ, USA) according to the standard method for the use of the caliper [42]. The upper arm of the caliper was lowered without compressing the abdomen and the arm of the caliper was placed at the level of L4–L5 under the participant. The reading on the vertical scale with a normal exhalation was noted in cm. BMI was calculated by dividing weight (kg) by height squared (m^2^). To measure blood pressure (BP), a mercury sphygmomanometer was used in the sitting position after a 10-min rest period. Two readings of systolic and diastolic BP were checked at 3-min intervals, and averages were recorded in the analysis. Blood samples were collected at baseline and after 12 weeks of study after a 12-h fast. Fasting blood glucose was reported using a glucose oxidase test method (LX-20, Beckman Coulter, Fullerton, CA, USA). Serum AST, ALT, and Cr were measured using a Toshiba TBA200FR biochemical analyzer (Toshiba Co. Ltd., Tokyo, Japan). 

### 4.6. Statistical Methods

The sample size of the study was calculated based on the research by Min et al. [43]. The estimated sample size was 45 patients per group for an 80% power to detect a difference in the mean investigator assessment score of 0.8, assuming a standard deviation of 1.2472 in the primary outcome variables and an alpha error of 5% with a 10% of drop-off rate. When a result of a test was unavailable, the last recorded data entry was included in the analysis (the last observation carried forward method). Efficacy analysis was conducted on both an intention to treat (ITT) basis on subjects that received at least one dose of SCP or placebo and that underwent at least one assessment post-baseline and per protocol (PP) only including data from subjects that completed the study protocol as planned. The Shapiro-Wilk’s test was employed to test the normality assumption. Intergroup comparisons of baseline characteristics and their changes at the 12th week of the trial were done using the two-sample *t*-test for continuous variables (or Mann-Whitney’s U test in case of valuables showing non-normal distributions) or the chi-square test for categorical variables (or Fisher’s exact test in case of valuables showing non-normal distributions). Intragroup comparisons were conducted using the paired *t*-test for continuous variables (or Mann-Whitney’s U test in case of valuables showing non-normal distributions). An analysis of covariance (ANCOVA) was performed to compare intergroup differences in outcomes after adjustment for physical activities and covariates that had shown a statistical significance between the groups at baseline. A *p*-value of less than 0.05 was considered statistically significant. SPSS version 22.0 (SPSS Statistics for Windows Version 22.0, Armonk, NY, IBM Corp) was employed for the analysis.

## Figures and Tables

**Table 1:** Baseline characteristics of the study subjects.

Variable	Intention to Treat Analysis			Per Protocol Analysis		
	CG (*n* = 45)	IG (*n* = 45)	*p* *	CG (*n* = 41)	IG (*n* = 40)	*p* *
Male (%)	8 (17.8)	9 (20.0)	0.788	8 (19.5)	8 (20.0)	0.956
Age (years)	40.8 ± 11.1	41.7 ± 9.7	0.750	41.1 ± 11.2	41.8 ± 9.9	0.817
BMI (kg/m^2^)	25.8 ± 1.9	25.5 ± 2.0	0.467	25.8 ± 1.9	25.4 ± 2.0	0.404
Alcohol user (%)	23 (51.1)	18 (40.0)	0.290	20 (48.8)	17 (42.5)	0.570
Smoker (%)	2 (4.4)	1 (2.2)	0.559	2 (4.9)	1 (2.5)	0.573
Calorie intake (kcal/day)	1660 ± 495	1594 ± 357	0.410	1667 ± 515	1601 ± 360	0.427
IPAQ (METs)	1151 (363–1726)	740 (33–2170)	0.813	1158 (396–1658)	903 (33–2655)	0.502

BMI, body mass index; IPAQ, international physical activity questionnaires; SAD, sagittal abdomen distance. Data was presented as mean ± standard deviation or number (%) except for IPAQ with median (interquartile). * By Chi-square test or two sample *t*-test except for IPAQ using Mann-Whitney’s U test.

**Table 2:** Comparison of changes in measurements in the intention to treat (ITT) population.

Variable	Observed Values			Changes from Baseline				
	CG (*n* = 45)	IG (*n* = 45)	*p* *	CG (*n* = 45)	*p* **	IG (*n* = 45)	*p* **	*p* *
Weight (kg)								
Baseline	68.0 ± 8.5	66.6 ± 8.5	0.364 ^1^					
At 12 weeks	68.7 ± 8.8	66.8 ± 8.8	0.228 ^1^	0.7 ± 1.9	0.018 ^4^	0.2 ± 1.3	0.183 ^4^	0.155 ^2^
SAD (cm)								
Baseline	19.6 ± 2.0	19.3 ± 1.6	0.226 ^1^					
At 12 weeks	19.5 ± 2.2	19.2 ± 1.8	0.433 ^2^	−0.1 ± 1.1	0.489 ^3^	−0.1 ± 1.0	0.701 ^4^	0.840 ^4^
BMI (kg/m^2^)								
Baseline	25.8 ± 1.9	25.5 ± 2.0	0.467 ^2^					
At 12 weeks	26.1 ± 2.2	25.6 ± 2.0	0.274 ^2^	0.3 ± 0.7	0.015 ^3^	0.1 ± 0.5	0.265 ^3^	0.153 ^1^
Body fat (%)								
Baseline	40.5 ± 4.7	41.2 ± 5.5	0.255 ^1^					
At 12 weeks	40.3 ± 4.4	40.6 ± 5.3	0.455 ^1^	−0.2 ± 1.4	0.364 ^3^	−0.6 ± 1.5	0.017 ^4^	0.226 ^1^
Fat mass (kg)								
Baseline	27.4 ± 4.0	28.2 ± 7.0	0.744 ^1^					
At 12 weeks	27.7 ± 4.0	27.0 ± 4.3	0.458 ^2^	0.3 ±1.4	0.154 ^3^	−1.2 ± 4.8	0.072 ^4^	0.025 ^1^
Lean mass (kg)								
Baseline	40.5 ± 7.0	39.8 ± 8.2	0.260 ^1^					
At 12 weeks	41.1 ± 7.0	39.8 ± 7.5	0.139 ^1^	0.6 ± 1.4	0.154 ^3^	−0.1 ± 4.0	0.011 ^4^	0.762 ^1^
Adiponectin (ug/mL)								
Baseline	3.95 ± 2.0	4.36 ± 2.1	0.314 ^1^					
At 12 weeks	4.23 ± 1.8	4.79 ± 2.3	0.456 ^1^	0.28 ± 0.7	0.003 ^4^	0.43 ± 1.48	0.007 ^4^	0.762 ^1^
Total Cholesterol (mg/dL)								
Baseline	199.4 ± 28.9	203.0 ± 33.6	0.592 ^2^					
At 12 weeks	200.6 ± 32.7	205.8 ± 32.6	0.448 ^2^	1.2 ± 24.5	0.753 ^3^	2.8 ± 17.9	0.292 ^3^	0.288 ^1^
Triglyceride (mg/dL)								
Baseline	128.3 ± 114.3	124.2 ± 88.4	0.741 ^1^					
At 12 weeks	138.0 ± 85.9	136.8 ± 102.1	0.710 ^1^	9.7 ± 95.0	0.009 ^4^	12.6 ± 51.2	0.095 ^4^	0.301 ^1^
HDL-Cholesterol (mg/dL)								
Baseline	55.0 ± 10.5	57.7 ± 13.7	0.290 ^2^					
At 12 weeks	54.6 ± 11.2	55.6 ± 12.8	0.490 ^1^	−0.4 ± 8.9	0.141 ^4^	−2.1 ± 5.9	0.020 ^3^	0.665 ^1^
LDL-Cholesterol (mg/dL)								
Baseline	118.8 ± 28.1	120.9 ± 26.9	0.711 ^2^					
At 12 weeks	119.1 ± 26.8	122.8 ± 27.5	0.529 ^2^	0.4 ± 23.8	0.739 ^3^	1.8 ± 17.1	0.474 ^3^	0.355 ^1^

BMI, body mass index; HDL, high density lipoprotein; LDL, low density lipoprotein; SAD, sagittal abdomen distance. Shapiro-Wilk’s test was employed for test of normality assumption * *p* values were compared within each group from baseline. ** *p* values were compared between groups. ^1^ *p* values were derived from Mann-Whitney’s U test. ^2^ *p* values were derived from independent *t* test. ^3^ *p* values were derived from paired *t* test. ^4^ *p* values were derived from Wilcoxon’s signed rank test.

**Table 3:** Changes in laboratory results related to safety in the ITT population.

Variable	Control (*n* = 45)			Collagen (*n* = 45)		
	Week 0	Week 12	*p*	Week 0	Week 12	*p*
AST (IU/L)	24.51 ± 7.9	20.93 ± 5.8	0.003	23.84 ± 10.0	24.87 ± 11.8	0.066
ALT (IU/L)	22.31 ± 13.3	20.76 ± 9.5	0.876	24.02 ± 19.7	25.44 ± 20.1	0.932
Cr (mg/dL)	0.69 ± 0.14	0.66 ± 0.1	0.016	0.71 ± 0.1	0.69 ± 0.1	0.812
Glucose (mg/dL)	91.67 ± 11.8	88.6 ± 9.7	0.033	90.07 ± 10.5	89.58 ± 12.6	0.244

AST, aspartate aminotransferase; ALT, alanine aminotransferase; Cr, creatinine. *p* values were compared within each group from baseline.
